# Proteomic response of *Escherichia coli* to a membrane lytic and iron chelating truncated *Amaranthus tricolor* defensin

**DOI:** 10.1186/s12866-021-02176-4

**Published:** 2021-04-12

**Authors:** Tessa B. Moyer, Ashleigh L. Purvis, Andrew J. Wommack, Leslie M. Hicks

**Affiliations:** 1 Department of Chemistry, University of North Carolina at Chapel Hill, 125 South Rd. CB#3290, Chapel Hill, NC 27599 USA; 2 Department of Chemistry, High Point University, High Point, NC USA

**Keywords:** Defensins, *Amaranthus tricolor*, Membrane lysis, Iron chelation, Iron reduction, *Escherichia coli*

## Abstract

**Background:**

Plant defensins are a broadly distributed family of antimicrobial peptides which have been primarily studied for agriculturally relevant antifungal activity. Recent studies have probed defensins against Gram-negative bacteria revealing evidence for multiple mechanisms of action including membrane lysis and ribosomal inhibition. Herein, a truncated synthetic analog containing the γ-core motif of *Amaranthus tricolor* DEF2 (Atr-DEF2) reveals Gram-negative antibacterial activity and its mechanism of action is probed via proteomics, outer membrane permeability studies, and iron reduction/chelation assays.

**Results:**

Atr-DEF2(G39-C54) demonstrated activity against two Gram-negative human bacterial pathogens, *Escherichia coli* and *Klebsiella pneumoniae*. Quantitative proteomics revealed changes in the *E. coli* proteome in response to treatment of sub-lethal concentrations of the truncated defensin, including bacterial outer membrane (OM) and iron acquisition/processing related proteins. Modification of OM charge is a common response of Gram-negative bacteria to membrane lytic antimicrobial peptides (AMPs) to reduce electrostatic interactions, and this mechanism of action was confirmed for Atr-DEF2(G39-C54) via an N-phenylnaphthalen-1-amine uptake assay. Additionally, in vitro assays confirmed the capacity of Atr-DEF2(G39-C54) to reduce Fe^3+^ and chelate Fe^2+^ at cell culture relevant concentrations, thus limiting the availability of essential enzymatic cofactors.

**Conclusions:**

This study highlights the utility of plant defensin γ-core motif synthetic analogs for characterization of novel defensin activity. Proteomic changes in *E. coli* after treatment with Atr-DEF2(G39-C54) supported the hypothesis that membrane lysis is an important component of γ-core motif mediated antibacterial activity but also emphasized that other properties, such as metal sequestration, may contribute to a multifaceted mechanism of action.

**Supplementary Information:**

The online version contains supplementary material available at 10.1186/s12866-021-02176-4.

## Background

Plant defensins comprise a well-known family of antimicrobial peptides (AMPs) broadly distributed across the plant kingdom [1]. Plant defensins can have a wide variety of functions including antibacterial activity, trypsin or α- amylase inhibition, and roles in plant development, but research has primarily focused on characterizing activity against economically important agricultural fungi [2, 3]. Antifungal defensins generally act via disruption of the plasma membrane and while mechanisms of action (MOA) vary, they often require binding to specific cell wall lipids before inducing secondary effects such as production of reactive oxygen species, disruption of Ca^2+^ signaling, or membrane lysis [4–6].

Although plant defensins’ antibacterial activity is less commonly characterized, several are known to target human and agricultural pathogens [2]. While antibacterial MOA remains poorly understood, recent studies have revealed contributions of membrane permeabilization and inhibition of transcription/translation in Gram-negative bacteria [7–9]. Resistance pathways include modifications to bacterial OM that decrease negative charge (lipid A modification and spermidine production) and mutations to ribosomes [7–9]. As some Gram-negative bacteria have developed resistance to many commercially available antibiotics [10], increasing understanding of susceptibility to antimicrobial peptides, such as plant defensins, could aid in the development of novel peptide-based therapeutics.

**Fig. 1:**
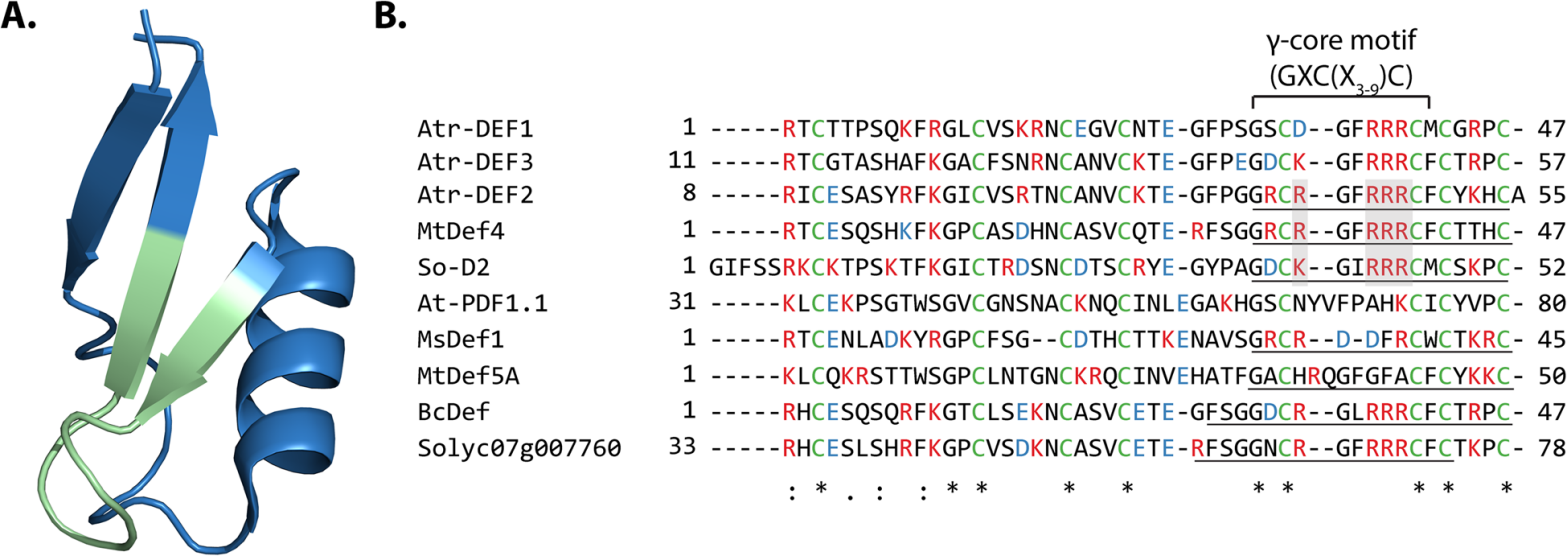
(**a**) 3D structure of *Medicago truncatula* defensin MtDef4 (PDB: 2LR3, figure generated in Pymol) [15] with its γ-core motif (GXCX_3-9_C, where X_n_ is the number of residues between cysteines) highlighted in green. (**b**) Alignment of Atr-DEF2 (predicted mature sequence) with other plant defensins including known sequence of Atr-DEF1 [16] and predicted mature sequence of Atr-DEF3. Underlined regions have been synthesized as γ-core motif analogs [8, 17, 18]. Cysteines forming disulfide bonds are green, basic residues are red, and acidic residues are blue. Conserved basic residues of membrane lytic γ-core motif analogs are shaded grey. Asterisks note fully conserved residues, two dots note positions with highly similar residues, and single dots note positions with weakly similar residues

Plant defensins are approximately 50 residues in length and contain eight conserved cysteine residues forming four disulfide bonds that increase stability against proteases, temperature and pH [11–14]. Plant defensins belong to the cis-defensin superfamily that have a characteristic antiparallel β-sheet bound to an α-helix by two disulfide bonds (Fig. 1a) [4, 15]. The significant length and complexity of defensins can hinder synthetic approaches to obtain sufficient quantities of defensins for extensive biological characterization. To circumvent this limitation and expedite the screening processes, previous studies have designed truncated defensins which are smaller (~ 1.2 kDa), less structurally complex (no disulfide bonds) and more synthetically tractable [8, 15, 17–20]. These analogs include the γ-core motif (GXCX_3-9_C, where X_n_ is the number of residues between cysteines) of target full length defensins [15, 17–20] (Fig. 1b). Previous activity comparisons between full length defensins and γ-core motif analogs suggest that the γ-core motif can exhibit approximately 10–40% of the activity of full length defensins and can serve as an effective proxy for full length peptides during initial activity screening and characterization [3].

Herein, we use a truncated γ-core motif analog from a yet previously uncharacterized *A. tricolor* defensin to explore activity against Gram-negative bacteria. We investigate the response of *E. coli* to Atr-DEF2(G39-C54) at a sub-lethal concentration revealing significant changes in the proteome associated with the OM and iron acquisition/processing. Validation assays confirm the capacity of Atr-DEF2(G39-C54) to perturb the outer membrane, reduce aqueous Fe^3+^, and chelate Fe^2+^ supporting the hypothesis that Atr-DEF2 is membrane lytic and initiates iron deficiency.

## Results

### Design of the synthetic truncated defensin

Previous in silico AMP predictions identified 20 putative defensins within the transcriptome of *A. tricolor*, nine of which contained a canonical γ-core motif (Figure S1) [21]. Three of these (denoted Atr-DEF1–3) included a highly basic γ-core motif similar to known active synthetic analogs (So-D2, BcDEF, Solyc07g007760, and MtDef4) (Fig. 1b) [8, 17, 18]. Atr-DEF2 displayed the most positive charge and thus was used to design a synthetic analog spanning the γ-core motif yielding Atr-DEF2(G39-C54) (Fig. 1b).

### Antibacterial activity

**Fig. 2:**
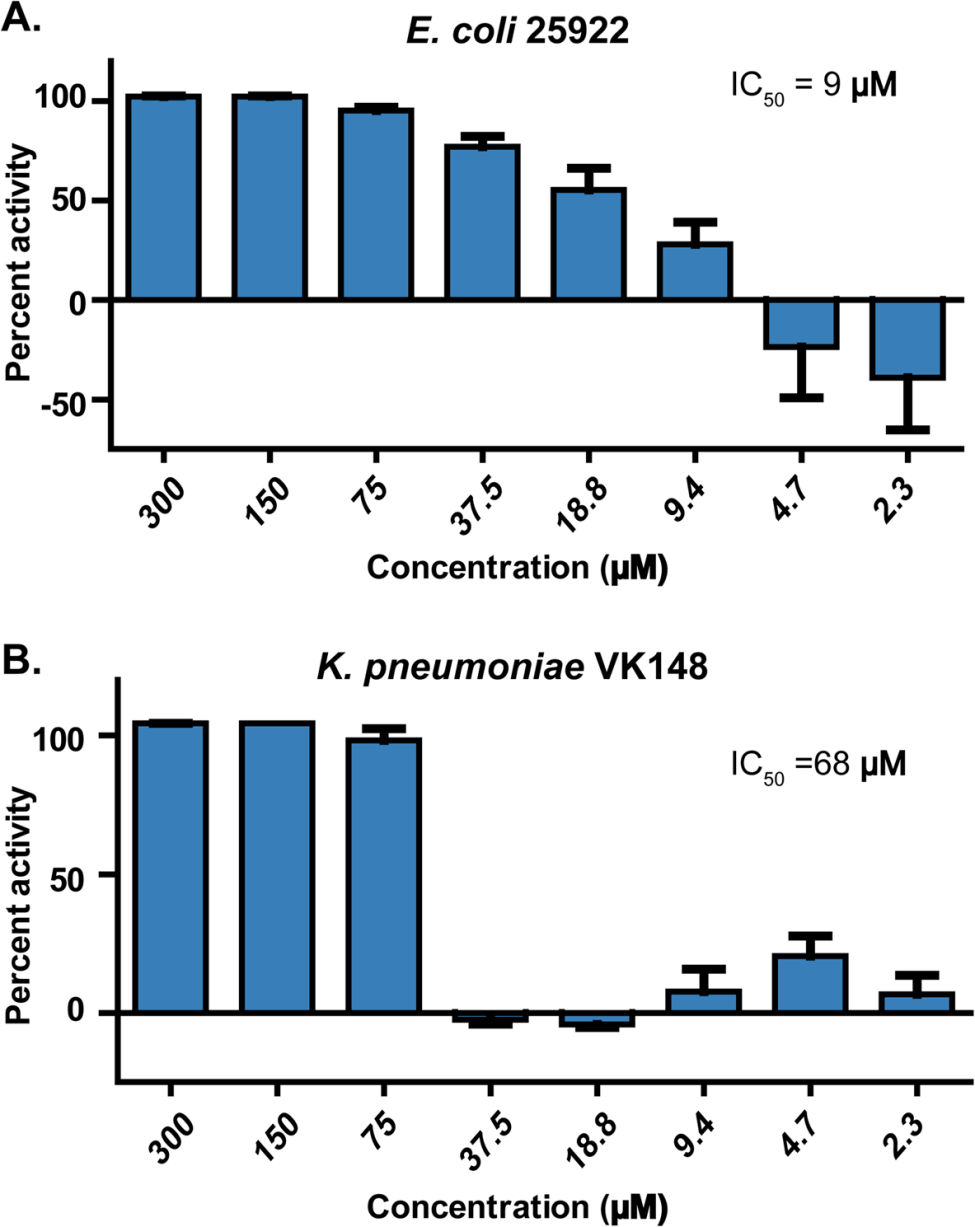
Antibacterial activity of Atr-DEF2(G39-C54) against (**a**) *E. coli* 25922 and (**b**) *Klebsiella pneumoniae* VK148

Atr-DEF2(G39-C54) was screened against Gram-negative *E. coli* 25922 (Fig. 2a) and *K. pneumoniae* VK148 (Fig. 2b). Both these pathogens belong to the *Enterobacteriaceae* family categorized as an urgent threat by the CDC [22]. Atr-DEF2(G39-C54) was active against *E. coli* (IC_50_ = 9 μM) (Fig. 2a and Supplementary Figure S2A) and *K. pneumoniae* (IC_50_ = 68 μM) (Fig. 2b and Supplementary Figure S2b).

### *E. coli* proteomic response to Atr-DEF2(G39-C54)

**Fig. 3:**
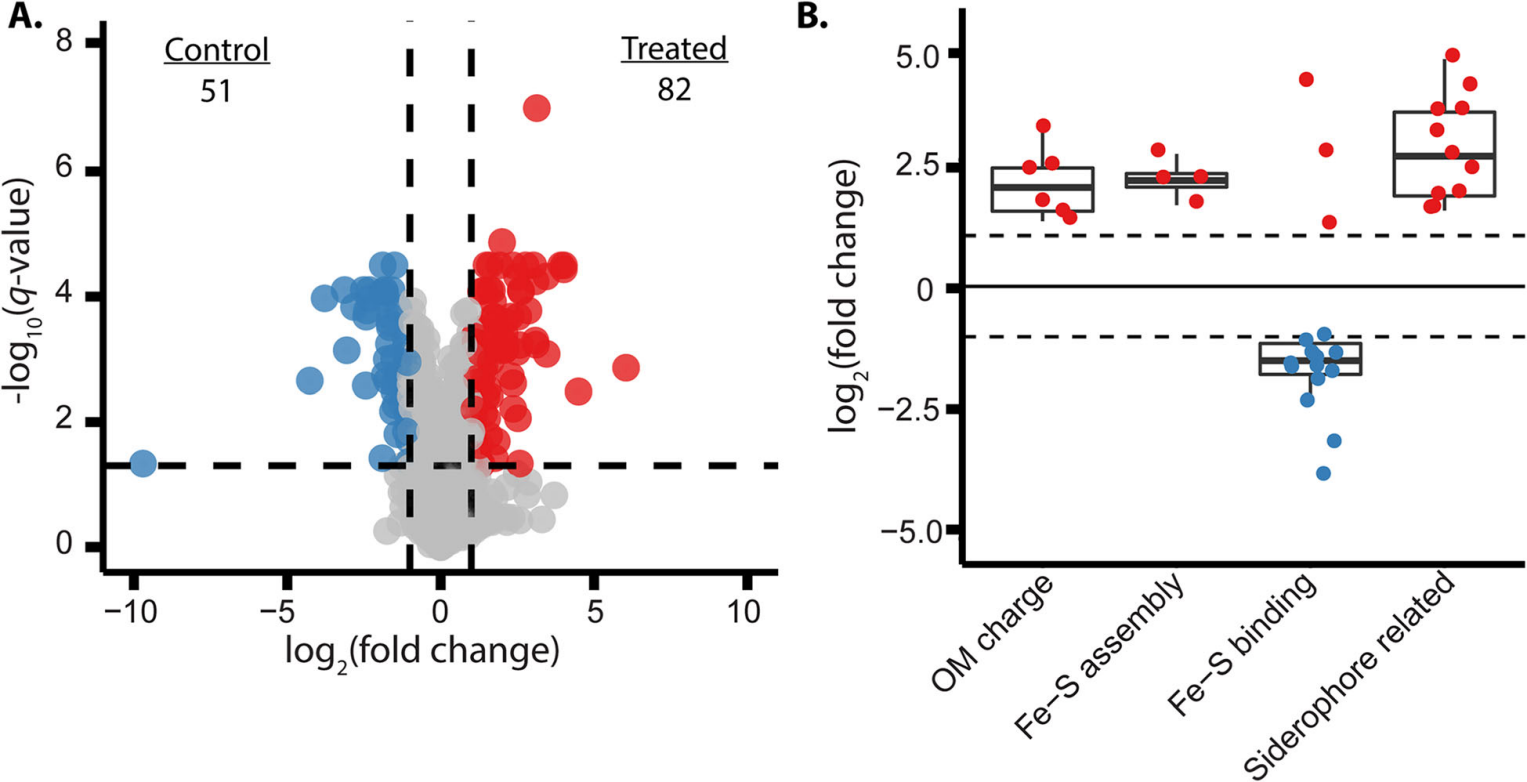
(**a**) Volcano plot visualizing global changes in *E. coli* proteome after treatment with Atr-DEF2(G39-C54). Fold change is defined as the average of treatment values divided by control values across four biological replicates. The two vertical black lines indicate log2 fold change values of 1 and − 1. The horizontal black line indicates an FDR-adjusted *P*-value of 0.05. In total, 1598 proteins were quantified. Upon treatment with Atr-DEF2(G39-C54) 51 proteins showed decreased abundance (blue) and 82 showed increased abundance (red). (B) Fold changes of proteins showing significantly altered abundance associated with modification of OM charge, Fe-S cluster assembly (GO:1990229), Fe-S cluster binding (GO:0051536), and siderophore synthesis (GO:0019290)/transportation

Label-free quantitative proteomics was used to investigate Atr-DEF2(G39-C54)-induced changes in the *E. coli* proteome to identify potential mechanisms of action or pathways to resistance. *E. coli* were treated with sub-inhibitory concentration of 37 μM Atr-DEF2(G39-C54) which reduced bacterial growth but did not result in complete lethality (Fig. 2a). Of the 1598 proteins quantified (Table S1), 82 showed increased abundance and 51 showed decreased abundance in the treatment versus the control (Fig. 3a). Proteomic trends were identified via manual parsing and gene ontology (GO) term enrichment analysis of proteins showing significantly altered abundance (Table S2). This analysis revealed that proteins associated with modification of outer membrane charge, Fe-S cluster assembly (GO:1990229), and siderophore synthesis (GO:0019290)/transport were increased in abundance, while those which utilize Fe-S clusters (GO:0051536) were generally decreased in abundance (Fig. 3b). These results suggested that Atr-DEF2(G39-C54) perturbed the OM and iron homeostasis of *E. coli*.

### Outer membrane permeabilization assay

**Fig. 4:**
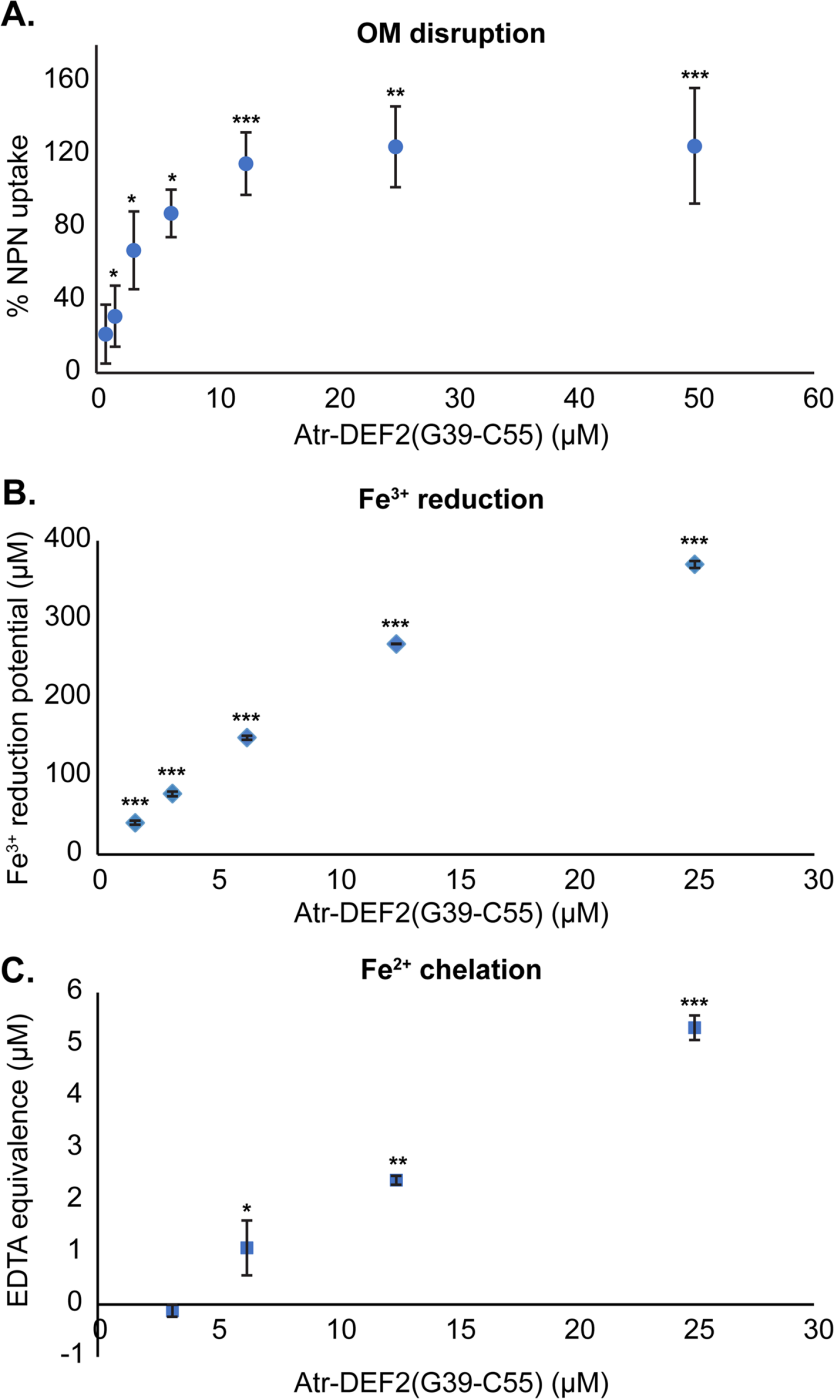
Atr-DEF2(G39-C54) validation experiments. (**a**) *E. coli* NPN uptake after treatment with Atr-DEF2(G39-C54). (**b**) Ferric reducing potential of Atr-DEF2(G39-C54) measured via FRAP assay. (**c**) Fe^2+^ chelating activity of Atr-DEF2(G39-C54) measured via decreased formation of UV-absorbing Fe^2+^-ferrozine complex and normalized to the chelating activity of EDTA. *P* value: *, *p* < 0.05 compared to negative control; **, *p* < 0.01; ***, *p <* 0.0001; *n* = 3

An N-phenylnaphthalen-1-amine (NPN)-based assay [23] was used to examine OM disruption by Atr-DEF2(G39-C54) as suggested by the proteomics results. Upon OM disruption, NPN partitions into the hydrophobic environment of the OM where it has increased fluorescence [23, 24]. Maximum NPN uptake was achieved at Atr-DEF2(G39-C54) concentrations of 12.5 μM and higher (Fig. 4a), indicating that the 37 μM concentration used for proteomics was sufficient to disrupt the OM of *E. coli*.

### Atr-DEF2(G39-C54)-Fe interactions

Proteomics suggested that Atr-DEF2(C39-C54) perturbs iron homeostasis. Thus, in vitro assays were used to confirm the capacity of Atr-DEF2(G39-C54) to reduce and/or chelate aqueous iron. The ability of Atr-DEF2(G39-C54) to induce iron-deficient growth conditions by reducing aqueous Fe^3+^ was tested using a ferric reducing antioxidant power (FRAP) assay [25]. This assay measures a decrease in absorbance as Fe^3+^ is reduced to Fe^2+^. Results revealed that Atr-DEF2(G39-C54) can reduce approximately equimolar Fe^3+^ at concentrations relevant to cell culture in Mueller Hinton broth (MHB) (~ 0.5–0.8 μM) [26] (Fig. 4b).

Chelation of Fe^2+^ by Atr-DEF2(G39-C54) was measured via a ferrozine-based spectroscopic assay in which the UV-absorbing ferrozine-Fe^2+^ complex is analyzed [27]. The presence of metal chelators in solution prevents complex formation, thereby decreasing absorbance. Ethylenediaminetetraacetic acid (EDTA) was used as a standard to normalize Fe^2+^ chelation by Atr-DEF2(G39-C54) to a well characterized chelator. Fe^2+^ chelation was observed at concentrations of 6.3 μM Atr-DEF2(G39-C54) and higher (Fig. 4c).

## Discussion

Defensins are a widely distributed class of antimicrobial peptides whose antibacterial activity is underexplored. Due to the size and complexity of full length defensins, synthetic defensin γ-core motif analogs offer a more tractable approach to examining the potential of predicted defensins from a range of host organisms. Herein, Atr-DEF2(G39-C54) designed from predicted *A. tricolor* defensin Atr-DEF2 exhibited Gram-negative antibacterial activity against human pathogens *E. coli* 25922 and *K. pneumoniae* VK148 (Fig. 2). *K. pneumoniae* VK148, a streptomycin- and rifampin-resistant mutant *K. pneumoniae* ATCC 43816 [28], was less sensitive to Atr-DEF2(G39-C54) than *E. coli* 25922. This decrease in activity is likely due to the presence of a thick anionic extracellular polysaccharide capsule which prevents cationic AMPs from reaching the bacterial membrane [29].

Atr-DEF2(G39-C54) was used to interrogate changes in the proteome of Gram-negative *E. coli* to defensin γ-core motif analogs via label-free proteomics. A sub-lethal concentration of Atr-DEF2(G39-C54) resulted in 133 proteins showing significantly altered abundances (Fig. 3a, Table S1). Trends within the proteins with significantly altered abundances suggested that *E. coli* cells were responding to OM and iron deficiency stress (Fig. 3b).

### Outer membrane perturbation

**Fig. 5:**
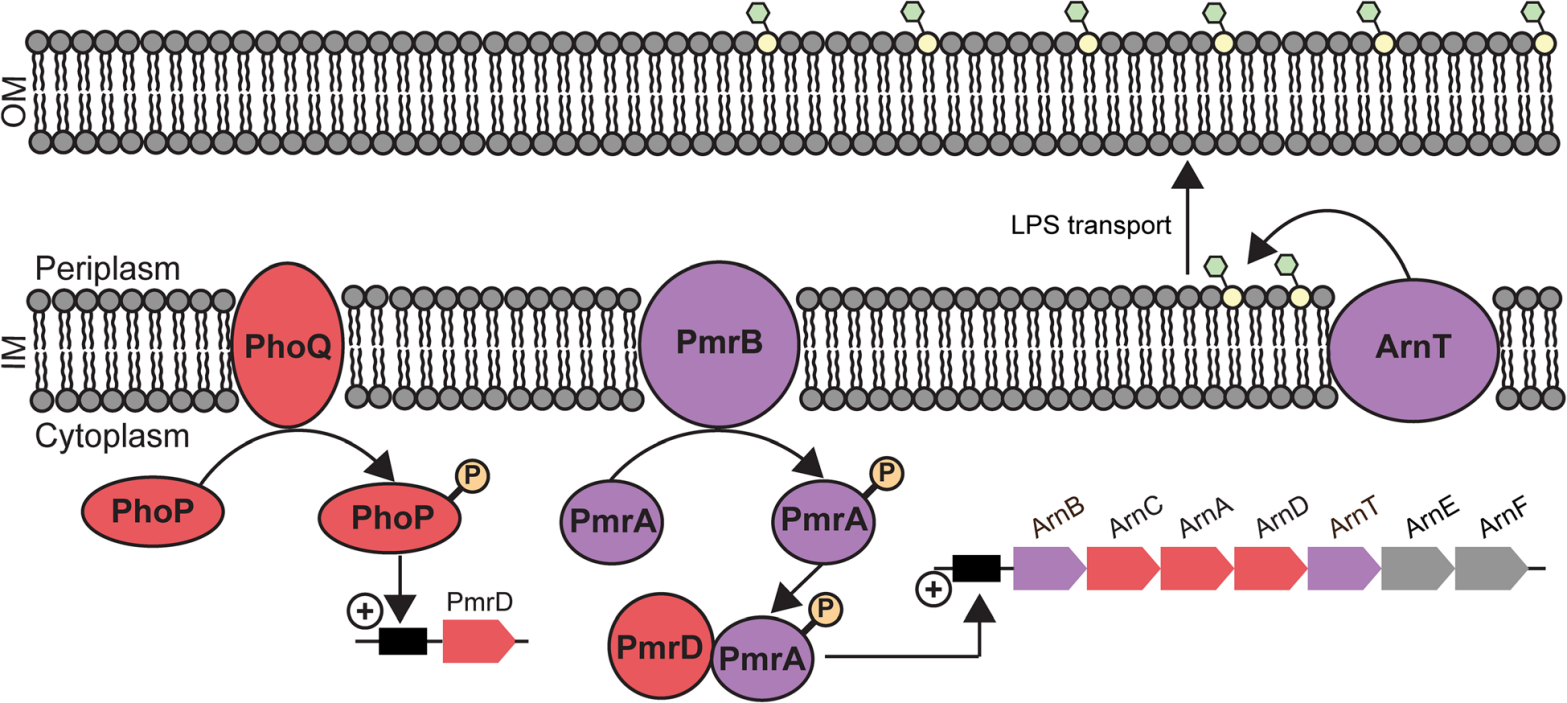
Decoration of lipid A in the outer membrane with L-Ara4N moieties is regulated by cross talk between two-component regulatory systems. Membrane lytic AMPs initiates the phosphorylation (orange) of PhoP by PhoQ activating translation of *pmrD*. PmrD protects PmrA (phosphorylated by PmrB) from dephosphorylation. Phosphorylated PmrA activates translation of the *arn* gene cluster. Proteins produced by the *arn* gene cluster synthesize L-Ara4N moieties (green) which are transferred by ArnT to lipid A (yellow). Modified lipopolysaccharides (LPS) are transported to outer membrane decreasing electrostatic interactions and inhibiting membrane lysis. Color scheme indicates proteins or protein products of genes whose abundance increased (red) or did not change (purple) after treatment with Atr-DEF2(G39-G54). Genes whose proteins products were not detected in either conditions are grey

A common adaptation of Gram-negative bacteria to membrane lytic antibiotics results in decreased electrostatic attraction between lipid A and cationic AMPs [30–33]. One pathway for charge reduction involves the addition of 4-amino-4-deoxy-L-arabinose (L-Ara4N) moieties to the phospholipid head group of lipid A, inhibiting electrostatic attraction of cationic AMPs [34] (Fig. 5). Lipid A aminoarabinose modification has been previously shown to impact the antibacterial activity of γ-core motif analogs of plant defensins Mtdef4 and So-D2 against Gram-negative bacteria [8]. Gram-negative *P. aeruginosa* strains with inhibited aminoarabinose modification pathways were more susceptible to Mtdef4/So-D2 γ-core motif analogs than the wild type strain [8]. So-D2, Mtdef4, and Atr-DEF2 all have similar highly charged γ-core motifs including a “RRR” repeat, (Fig. 1b) suggesting that high net charge enables membrane/peptide electrostatic interactions.

Six proteins within the lipid A aminoarabinose modification pathway showed increased abundance in Atr-DEF2(G39-C54) treated *E. coli* (Fig. 5, Table S1). This modification is mediated by the *arn* gene cluster and controlled via crosstalk between two signal regulatory systems transcriptional regulatory protein PhoP (P23837, FC: 4.8) / Sensor protein PhoQ (P23837, FC:8.6) and transcriptional regulatory protein PmrA (alternative name: BasR, A0A0H2VDK2) / Sensor protein PmrB (alternative name: BasS, A0A0H2VD68) [31–33, 35, 36]. These two histidine kinase/response regulator pairs are connected by PhoP activated signal transduction protein PmrD (P37590, FC: 5.1) which blocks dephosphorylation of PmrA [33] (Fig. 5, Table S1). The increase in abundance of these proteins suggests that *E. coli* is enhancing signaling related to OM stress response. Furthermore, bifunctional polymyxin resistance protein ArnA (Q8FFM1, FC: 2.7), undecaprenyl-phosphate 4-deoxy-4-formamido-L-arabinose transferase ArnC (Q8FFM2, FC: 3.1), and probable 4-deoxy-4-formamido-L-arabinose-phosphoundecaprenol deformylase ArnD (Q8FFM0, FC: 2.4) which are *arn* gene cluster protein products that catalyze the biosynthesis of L-Ara4N are also increased in abundance (Fig. 5, Table S1). This indicates that *E. coli* are masking OM negative charge with L-Ara4N moieties to prevent membrane lysis with Atr-DEF2(G39-G54). Thus, Atr-DEF2(G39-C54) was screened for the ability to disrupt membranes using an NPN-uptake assay (Fig. 4a). These results indicated that the sub-lethal 37 μM concentration of Atr-DEF2(G39-C54) used for the proteomic analysis would have been sufficient to perturb the OM of *E. coli* and contribute to the adaptive responses observed in the proteome.

### Iron-deficient environment

Transition metal sequestration is a known contributor to the activity of AMPs by limiting the availability of important inorganic cofactors and could account for the proteomic changes in iron acquisition, processing, and use [37]. Several plant defensins are known to chelate metal ions to confer heavy metal tolerance [38–40]. Although metal sequestration has not been previously reported as a direct antibacterial mechanism of action of plant defensins, AtPDF1.1 from *Arabidopsis thaliana* indirectly induces antibacterial resistance by chelating Fe^2+^ and triggering the ethylene signaling pathway [9].

**Fig. 6:**
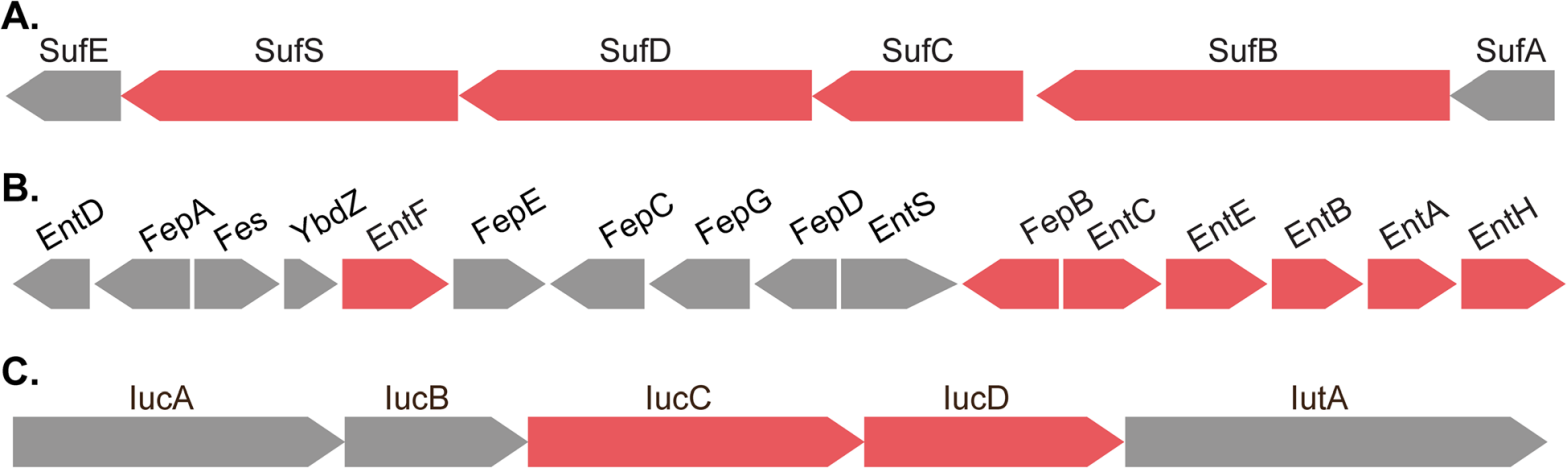
(**a**) Components of the sulfur formation (SUF) pathway gene cluster (as annotated in *E. coli* K-12 substr. MG1655 NCBI Accession: NC_000913), (**b**) enterobactin gene cluster (as annotated in *E. coli* K-12 substr. MG1655 NCBI Accession: NC_000913), and (**c**) aerobactin biosynthetic gene cluster (*E. coli* plasmid pVM01 NCBI Accession: NC_010409). Color scheme indicates genes whose protein products showed increased abundance (red) after treatment with Atr-DEF2(G39-C54) or where not detected in either condition (grey)

Initiation of iron deficiency by Atr-DEF2(G39-C54) was the second major trend to emerge from the proteomics analysis. Gene Ontology analysis revealed that several identifiers associated with Fe-S cluster assembly pathways (GO:1990229) or that bind Fe-S cluster cofactors (GO:0051536) were over represented in the proteomic changes induced by Atr-DEF2(G39-C54) (Table S2). Fe-S complexes are metal cofactors required for the function of a variety of essential processes (e.g. respiration and DNA repair) and can be assembled by *E. coli* via either the iron-sulfur cluster (ISC) or the sulfur formation (SUF) pathways [41]. The SUF pathway in *E. coli* is associated with Fe-S cluster assembly during iron deficiency or oxidative stress [41]. Upon treatment with Atr-DEF2(G39-C54), cysteine desulfurase SufS (Q8FH54, FC: 3.0), Fe-S cluster assembly protein SufD (A0A0H2V7L8, FC: 4.3), probable ATP-dependent transporter SufC (A0A0H2V7V5, FC: 4.2), and Fe-S cluster assembly protein SufB (A0A0H2V9X7, FC: 6.2) showed increased abundance (Fig. 6A, Table S1). All these proteins participate in the SUF pathway, suggesting the cell is adapting Fe-S assembly processes to an iron-limited environment.

Furthermore, fourteen proteins which use Fe-S cluster cofactors showed decreased abundance (FC: 0.07–0.49, Table S1) after treatment constituting a general trend towards reduction of Fe-S cluster utilization (Fig. 3b) and an overrepresentation within the decreasing proteins (Table S2). Eleven of the decreasing Fe-S cluster binding proteins were also associated with the GO term “generation of precursor metabolites and energy” (GO:0006091, Table S2) suggesting that iron limitation is causing changes to energy metabolism. For example, Fe-S binding proteins fumarate reductase iron-sulfur subunit FrdB (P0AC48, FC: 0.5), which converts fumarate to succinate during the final step of anaerobic respiration [42], and quinolinate synthase A NadA (Q8FJS7, FC: 0.5), which participates in NAD synthesis catalyzing a reaction between aminoaspartate with dihydroxyacetone phosphate [43], both showed decreased abundance after treatments. Previous studies reported decreases in iron-rich energy metabolism proteins upon analysis of the iron-deficient *E. coli* proteome [44, 45]. It is hypothesized this indicates a shift to pathways which require fewer Fe-S cofactors [44, 45].

Changes to siderophore-based iron acquisition processes also suggested a proteomic adaptation to iron deficiency. Siderophores are secondary metabolites which bind and import extracellular Fe^3+^ so that it can be incorporated into biomolecules [46]. Biosynthetic proteins which produce the siderophores enterobactin and aerobactin were increasing after treatment with Atr-DEF2(G39-G54). Enterobactin and aerobactin, produced by the *ent* and *iuc* gene clusters respectively (Fig. 6b and c), are known to be induced during iron-limited conditions to increase capacity of *E. coli* to scavenge for environmental iron [46, 47]. A total of seven *ent/iuc* protein products (3-dihydro-2,3-dihydroxybenzoate dehydrogenase EntA (A0A0H2V770, FC: 22.5), Isochorismatase EntB (A0A0H2V551, FC: 6.0), Isochorismate synthase EntC (A0A0H2V5R9, FC: 10.9), enterobactin synthetase component E EntE (A0A0H2V534, FC: 8.1), enterobactin synthetase component F entF (A0A0H2V5R2, FC: 4.9), IucA protein (A0A0H2VD46, FC: 3.5), and IucD protein (A0A0H2VC61, FC: 2.9)) showed increased abundance after treatment (Fig. 6 and Table S1). In addition to siderophore synthesis proteins, three siderophore transportation proteins showed increased abundance, including biopolymer transport proteins ExbB (A0A0H2VAW2, FC: 3.4) and ExbD (P0ABV3, FC: 2.8) (Table S1). ExbB and ExbD associate with TonB in the inner member to form an energy transducing complex which drives outer and inner membrane transportation of siderophores [48]. These proteins are regulated by the Fe^2+^-Fur repressor complex and are induced under iron-deplete growth conditions [48]. Together, these trends suggest that *E. coli* is increasing production of enterobactin siderophores in response a change in iron availability caused by Atr-DEF2(G39-C54).

In vitro assays were conducted to confirm the ability of Atr-DEF2(G39-C54) to perturb aqueous iron concentrations relevant to bacterial culture [26]. First, a FRAP assay revealed that Atr-DEF2(G39-C54) can reduce water-soluble Fe^3+^ to insoluble Fe^2+^ (Fig. 4b). Reduction of Fe^3+^ by Atr-DEF2(G39-C54) would make iron less bioavailable for scavenging and import via siderophores such as enterobactin which bind Fe^3+^ exclusively. Although Fe^3+^ is the oxidation state of iron that is scavenged by *E. coli* from the environment, Fe^2+^ is required for incorporation into biomolecules. Chelation of Fe^2+^ by Atr-DEF2(G39-C54) was then shown via decreased formation of a UV-absorbing ferrozine-Fe^2+^ complex [27] (Fig. 4c). Combined, these finding support the hypothesis that Atr-DEF2(G39-C54) reduces Fe^3+^ and chelates Fe^2+^, resulting in iron deficient growth conditions and limiting the availability of iron-based cofactors.

## Conclusions

Plant defensins are a well-studied family of AMPs whose antibacterial activity has been largely unexplored. Here, a truncated analog designed from the γ-core motif of a predicted *A. tricolor* defensin [Atr-DEF2(G39-C54)] demonstrated robust activity against both Gram-negative *E. coli* and *K. pneumoniae* pathogens and was utilized to investigate antibacterial mechanism of action. The response of *E. coli* to sub-lethal pressure by Atr-DEF2(G39-C54) revealed proteome changes associated with outer membrane modification and iron acquisition/processing. The capacity of Atr-DEF2(G39-C54) to disrupt the outer membrane and interact with aqueous iron was confirmed via an outer membrane permeability study and iron reduction/chelation assays. These results further support that membrane lysis plays an important role in defensin antibacterial mechanism of action mediated by the γ-core motif. Furthermore, it suggests that iron sequestration may contribute to antibacterial activity decreasing the availability of important inorganic cofactors.

## Materials and methods

### Design and synthesis of truncated defensins

Defensins previously predicted from the 1000 Plant Project transcriptome (1000 Plant Project sample ID: XSSD) [49, 50] of *Amaranthus tricolor* using Cysmotif Searcher (https://github.com/fallandar/cysmotifsearcher) [21, 51] were aligned with ClustalOmega (1.2.4) [52]. The synthesis of Atr-DEF2(G39-C54) was accomplished using a semi automated flow chemistry instrument built in-house [53, 54]. In brief, synthesis was performed using 200 mg of Fmoc-Cys (Trt)-2-chlorotrityl resin (0.77 mmol/g, 200–400 mesh). A solution of 20% piperidine in DMF was used for Fmoc deprotection. Following thorough washing of the resin with DMF, 1 mmol of each Fmoc amino acid was added as a 0.38 M HBTU in DMF (2.5 mL) solution with 450 mL of DIPEA (or 250 mL with His, Cys, or Trp).

Following synthesis completion, the resin-bound peptide was cleaved with a 10-min incubation at 60 °C with gentle stirring using a TFA/EDT/TIPS/H_2_O (94:2.5:2.5:1.0) cleavage mixture (20 mL). The crude cleaved peptide solution was filtered and the volume was reduced to ca. 5 mL under a stream of N_2_ gas. The crude peptide was then precipitated with diethyl ether at 4 °C. Following centrifugation (3000 rpm, 4 °C, 10 min) to collect the crude peptide precipitate, the pellet was dried in vacuo to deliver Atr-DEF2(G39-C54). The peptide was further purified by semi preparative HPLC (10–60% B over 20 min, 5 mL/min).

### IC_50_ assay

*E. coli* 25922 and *K. pneumoniae* VK148 assays were conducted in triplicate as previously described [54]. Atr-DEF2(G39-C54) is prone in precipitation when added to bacterial culture. Bioassay plate wells each contained 20 μL 1x Mueller–Hinton broth (MHB, 21 g/L, Difco), 10 μL 2x MHB (42 g/L), 10 μL bacterial culture, and 10 μL peptide samples. Controls consisted of ampicillin (*E. coli* positive control, 200 μg/mL), erythromycin (*K. pneumoniae* positive control, 200 μg/mL), and sterile water (negative control). Plates were incubated at 37 °C, 250 rpm for 4 h. Resazurin was added to each well to a final concentration of 1 mM and incubated for approximately 1 h. Cell viability was quantified using an excitation wavelength of 544 nm and emission wavelength of 590 nm. Bacterial cultures were challenged with a 2-fold dilution series of Atr-DEF2(G39-C54) ranging from a final concentration of 300 μM - 2.3 μM. Atr-DEF2(G39-C54) is prone in precipitation when added to bacterial culture and can skew optical density measurements. Percent activity was calculated using resazurin fluorescence as previously described [55]. IC_50_ curves were fitted using GraphPad Prism 5.

### Growth and harvest of *E. coli* treated with Atr-DEF2(G39-C54)

All culturing was conducted at 37 °C with shaking (250 rpm). *E. coli* 25922 was inoculated in 5 mL MHB and grown for 16 h. The overnight culture was used to inoculate three 5 mL MHB cultures to a final OD_600_ of 0.25. After 1 h, *E. coli* cultures were homogenized and the homogenate was used to generate eight 0.1 OD_600_ 1 mL cultures. Then, 1 mL cultures were treated with 37 μM Atr-DEF2(G39-C54) or water such that four biological replicates were performed for each condition. After 3 h, each replicate was harvested via centrifugation. Cell pellets were flash frozen in liquid nitrogen and stored at − 80 °C until further analysis.

### Protein extraction

Cell pellets were resuspended in 1.8 mL 100 mM Tris-HCl (pH 8) and lysed by sonicating for 2 min at 200 cycles/burst, with 150 W power and a 13% duty cycle using an E220 focused ultra-sonicator (Covaris, http://covaris.com/). Cell lysate was incubated for 30 min with 9 mL of chilled 100 mM ammonium acetate in methanol (− 20 °C) and then centrifuged at 3220 rcf for 10 min (4 °C) to pellet protein precipitate. Supernatant was discarded and the protein pellet dried for 20 min to remove any remaining methanol. Pellets were resuspended in 300 μL 4 M urea 100 mM Tris-HCl pH 8. Proteins concentration was determined using CB-X assay (G-Biosciences, St. Louis, MO).

### Protein reduction, alkylation, and digestion

50 μg aliquots of each replicate were reduced with 10 mM dithiothreitol (30 min, room temperature, dark) and alkylated with 30 mM iodoacetamide (45 min, room temperature, dark). Reduced and alkylated samples were precipitated in chilled acetone for 30 min (− 20 °C). Supernatant was discarded and protein pellets were dried under a stream of N_2(g)_. Pellets were resuspended in 400 μL 4 M urea 100 mM Tris-HCl (pH 8). Tryptic digestion (Trypsin Gold, Promega) was performed at an enzyme:protein ratio of 1:50 and incubated at 25 °C for 16 h with 850 rpm shaking. Digestion was quenched by acidifying with 10% trifluoracetic acid (pH < 3). Samples were desalted via C_18_ SepPak (50 mg, Waters) prior to further analysis.

### LC-MS/MS data acquisition

Samples were analyzed as previously described using an Acquity M-class UPLC system (Waters, Milford, MA, USA) coupled to a Q Exactive HF-X Hybrid Quadrupole-Orbitrap mass spectrometer (Thermo Scientific, Waltham, MA, USA) equipped with a Nanospray Flex source operated positive polarity mode [56]. Injections (4 μL) were made to a Symmetry C18 trap column (100 Å, 5 μm, 180 μm × 20 mm; Waters) and then separated on a HSS T3 C18 column (100 Å, 1.8 μm, 75 μm × 250 mm; Waters) resulting in an average peak width of 30 s. Data was acquired using a top 20 data-dependent acquisition mode with an isolation window of 1.5 *m/z*. Survey scans were collected with a scan range of 350–2000 *m/z*, 120,000 resolving power, an AGC target of 1 × 10^6^, and maximum injection time of 50 ms. Precursor ions were selected (isolation window of 1.5 *m/z*) for higher-energy collisional dissociation (HCD) collecting spectra with a scan range of 200–2000 *m/z,* resolving power of 30,000, AGC target of 3 × 105 and a maximum injection time of 100 ms. The total duty cycle of this method is 2.05 s producing approximately 15 survey scans across a chromatographic peak.

### Database searching and label-free quantification

LC-MS/MS data were processed as follows for area under the curve label-free quantitation. Acquired spectral files (*.raw) were imported into Progenesis QI for proteomics (Nonlinear Dynamics, version 2.0; Northumberland, UK). Peak picking sensitivity was set to maximum of five and a reference spectrum was automatically assigned. Total ion chromatograms (TICs) were then aligned to minimize run-to-run differences in peak retention time. Each sample received a unique factor to normalize all peak abundance values resulting from systematic experimental variation. A combined peak list (*.mgf) containing the top 25 fragmentation spectra for each *m/z* was exported for peptide sequence determination and protein inference by Mascot (Matrix Science, version 2.5.1; Boston, MA, USA). Database searching was performed against the *Escherichia coli* O6:H1 UniProt proteome (https://www.uniprot.org/proteomes/UP000001410, 5336 entries) and sequences for common laboratory contaminants (https://www.thegpm.org/cRAP/, 116 entries). Searches of MS/MS data used a trypsin protease specificity with the possibility of two missed cleavages, peptide/fragment mass tolerances of 15 ppm/0.02 Da, and variable modifications of protein N-terminus acetylation, and methionine oxidation. Alkylation of Cys with IAM (carbamidomethylcysteine) was set as a fixed modification. Significant peptide identifications above the identity or homology threshold were adjusted to less than 1% peptide FDR using the embedded Percolator algorithm [57] and imported to Progenesis for peak matching. Identifications with a Mascot score less than 13 were removed from consideration in Progenesis before exporting both “Protein Measurements” from the “Review Proteins” stage.

### Data analysis and statistics

For LC-MS/MS-based proteomics, data were parsed using custom scripts written in R for pre-processing and statistical analysis (https://github.com/hickslab/QuantifyR). Leading protein accessions were considered from the “Protein Measurements” data and kept if there were ≥ 2 shared peptides and ≥ 1 unique peptide assigned. Proteins were removed if there was not at least one condition with 3/4 nonzero values across the Progenesis-normalized abundance columns. Values were log_2_-transformed and we applied a conditional imputation strategy using the imp4p package [58], where conditions with at least one nonzero value had missing values imputed using the *impute.rand* function with default parameters. For cases where a condition had only missing values, the *impute.pa* function was used to impute small numbers centered on the lower 2.5% of values in each replicate. Statistical significance was determined using a two-tailed, equal variance *t*-test and the method of Benjamini- Hochberg (BH) was used to correct *p*-values for multiple comparisons [59]. Fold change was calculated by the difference of the mean abundance values between conditions being compared. Only observations with FDR-adjusted *p* < 0.05 and log_2_-transformed fold change +/− 1.0 were considered significantly different.

### Gene ontology enrichment analysis

Significantly increasing and decreasing proteins were analyzed for over/under-represented gene ontology (GO) terms using the Panther Gene Ontology enrichment analysis tool (v16.0, http://www.pantherdb.org/) [60]. Individual lists of increasing and decreasing proteins were submitted for analysis using all identified *E. coli* proteins as the reference data set.

### Outer membrane lysis assay

The capacity of Atr-DEF2(G39-C54) to disrupt the OM of *E. coli* was measured using an 1-N-phenylnapthylamine (NPN)-uptake assay [23]. 5 mL cultures of *E. coli* 25922 in MHB were incubated for 16 h at 37 °C with shaking (250 rpm). The overnight culture was used to inoculate three 5 mL MHB cultures to a final OD_600_ of 0.25 and incubated for an additional hour (37 °C, 250 rpm). *E. coli* cells were pelleted via centrifugation (1000 rcf, 5 min) and washed with 5 mL 5 mM HEPES (pH 7.4) buffer containing 5 mM glucose. Cells were again pelleted, discarding the supernatant and resuspending in 5 mL 5 mM HEPES (pH 7.4) buffer containing 5 mM glucose. NPN (0.5 mL, 0.110 mM resuspended in acetone) was added to the cell solution and incubated for 30 min (dark, room temperature). Cell solution (40 μL) and sample (10 μL) were combined in a 96-well plate and centrifuged briefly (1000 rcf) before measuring fluorescence (350 nm ex, 420 nm em). Samples were composed of a two-fold dilution series of Atr-DEF2(G39-C54) ranging from 250 to 2 μM (working concentration 50–0.4 μM), 50 μg/mL polymyxin B (working concentration 10 μg/mL, positive control), and MilliQ water (negative control). Each sample was assayed in triplicate. NPN uptake was calculated as follows, where F_obs_ is the fluorescence of the peptide sample, F_o_ is the fluorescence of the negative control and F_100_ is the fluorescence of the positive control:

$$ \mathrm{NPN}\ \mathrm{uptake}=\left(\left({\mathrm{F}}_{\mathrm{obs}}-{\mathrm{F}}_0\right)/\left({\mathrm{F}}_{100}-{\mathrm{F}}_0\right)\right)\times 100 $$

### FRAP assay

Fe^3+^ reduction was measured using a QuantiChrom FRAP assay kit (BioAssay Systems, Hayward, CA) per manufacturer protocol for 96-well plate format. Atr-DEF2(G39-C54) was assayed in a two-fold dilution series such that working concentrations ranged from 6.3–0.8 μM.

### Fe (II) chelation assay

Fe^2+^chelation assay was adapted from Santos et al. [27] Atr-DEF2(G39-C54) and EDTA samples were assayed in two-fold dilution series such that their working concentrations ranged from 0 to 13 μM. Sample (25 μL, 0–67.6 μM), water (80 μL), and (NH_4_) Fe (SO_4_)_2_ (0.3 mM, 10 μL) were incubated in a 96-well plate for 5 min at room temperature. Ferrozine (15 μL, 0.8 mM) was added and incubated for 15 min at room temperature before measuring the absorbance of each well at 562 nM. EDTA standards yielded a linear calibration curve which was used to determine the EDTA equivalence of Atr-DEF2(G39-C54) as follows, where *A* is the absorbance of each sample; *b* is the y intercept of the EDTA calibration curve, and *m* is the slope of the calibration curve:

$$ EDTA\ equivalence=\frac{A-b}{m} $$

## Supplementary Information

**Additional file 1: Tables S1 and S2.** Tables detailing global proteomic identifications (Table S1) and Panther Gene Ontology enrichment analysis (Table S2).

**Additional file 2: Figures S1 and S2.** Figures illustrating alignment of defensins predicted from the transcriptome of *A. tricolor* (Fig. S1) and the IC50 of Atr-DEF2(G39-C54) against *E. coli* 25922 and *K. pneumoniae* VK148 (Fig. S2).

## Data Availability

The datasets generated and/or analyzed during the current study are available the supplemental files of this published article and the Proteomics Identifications Database (PRIDE) repository, [PXD024138, 10.6019/PXD024138] [61].

## Abbreviations

AMP: Antimicrobial peptide
MOA: Mechanism of action
OM: Outer membrane
IC_50_: Inhibitory concentration 50%
CDC: Centers for Disease Control
GO: Gene ontology
NPN: N-phenylnaphthalen-1-amine
L-Ara4N: 4-amino-4-deoxy-L-arabinose
So-D2: *Spinacia oleracea* defensin 2
MtDef4: *Medicago truncatula* defensin 4
Atr-DEF1: *Amaranthus tricolor* defensin 1
Atr-DEF2: *Amaranthus tricolor* defensin 2
Atr-DEF2(G39-C54): Synthetic analog of Atr-DEF2
BcDEF: *Brugmansia x candida* defensin
MHB: Mueller Hinton broth
AtPDF1.1: *Arabidopsis thaliana* defensin 1.1
FRAP: Ferric reducing antioxidant power
IAM: Iodoacetamide
FC: Fold change
Fmoc-Cys (Trt)-2-chlorotrityl resin: Fmoc-S-trityl-L-cysteine-2-chlorotrityl resin
Fmoc: Fluorenylmethyloxycarbonyl
DMF: Dimethylformamide
HBTU: 2-(1H-benzotriazol-1-yl)-1,1,3,3-tetramethyluronium hexafluorophosphate
DIPEA: N,N-diisopropylethylamine
TFA: Trifluoroacetic acid
EDT: Ethanedithiol

## References

[1] Lay F, Anderson M (2005). Defensins - components of the innate immune system in plants. Curr Protein Pept Sci.

[2] Sathoff AE, Samac DA (2019). Antibacterial activity of plant Defensins. Mol Plant-Microbe Interact.

[3] Sathoff AE, Velivelli S, Shah DM, Samac DA (2019). Plant defensin peptides have antifungal and antibacterial activity against human and plant pathogens. Phytopathology..

[4] Parisi K, Shafee TMA, Quimbar P, van der Weerden NL, Bleackley MR, Anderson MA (2019). The evolution, function and mechanisms of action for plant defensins. Semin Cell Dev Biol.

[5] Spelbrink RG, Dilmac N, Allen A, Smith TJ, Shah DM, Hockerman GH (2004). Differential antifungal and Calcium Channel-blocking activity among structurally related plant Defensins. Plant Physiol.

[6] Poon IKH, Baxter AA, Lay FT, Mills GD, Adda CG, Payne JAE (2014). Phosphoinositide-mediated oligomerization of a defensin induces cell lysis. Elife..

[7] Velivelli SLS, Islam KT, Hobson E, Shah DM (2018). Modes of action of a bi-domain plant Defensin MtDef5 against a bacterial pathogen Xanthomonas campestris. Front Microbiol.

[8] Sathoff AE, Lewenza S, Samac DA (2020). Plant defensin antibacterial mode of action against Pseudomonas species. BMC Microbiol.

[9] Hsiao PY, Cheng CP, Koh KW, Chan MT (2017). The Arabidopsis defensin gene, AtPDF1.1, mediates defence against Pectobacterium carotovorum subsp. carotovorum via an iron-withholding defence system. Sci Rep.

[10] Rossolini GM, Arena F, Pecile P, Pollini S (2014). Update on the antibiotic resistance crisis. Curr Opin Pharmacol.

[11] Kovaleva V, Bukhteeva I, Kit OY, Nesmelova IV (2020). Plant defensins from a structural perspective. Int J Mol Sci.

[12] Tam JP, Wang S, Wong KH, Tan WL (2015). Antimicrobial peptides from plants. Pharmaceuticals..

[13] Vriens K, Cammue BPA, Thevissen K (2014). Antifungal plant defensins: mechanisms of action and production. Molecules..

[14] Goyal RK, Mattoo AK, Epand RM (2016). Plant antimicrobial peptides. Host defense peptides and their potential as therapeutic agents.

[15] Sagaram US, Pandurangi R, Kaur J, Smith TJ, Shah DM (2011). Structure-activity determinants in antifungal plant Defensins MsDef1 and MtDef4 with different modes of action against Fusarium graminearum. PLoS One.

[16] Moyer TB, Allen JL, Shaw LN, Hicks LM (2021). Multiple Classes of Antimicrobial Peptides in Revealed by Prediction, Proteomics, and Mass Spectrometric Characterization. J Nat Prod.

[17] Rigano MM, Romanelli A, Fulgione A, Nocerino N, D’Agostino N, Avitabile C (2012). A novel synthetic peptide from a tomato defensin exhibits antibacterial activities against *Helicobacter pylori*. J Pept Sci.

[18] Kaewklom S, Wongchai M, Petvises S, Hanpithakphong W, Aunpad R (2018). Structural and biological features of a novel plant defensin from Brugmansia x candida. PLoS One.

[19] Aerts AM, François IEJA, Meert EMK, Li QT, Cammue BPA, Thevissen K (2007). The antifungal activity of RsAFP2, a plant defensin from Raphanus sativus, involves the induction of reactive oxygen species in Candida albicans. J Mol Microbiol Biotechnol.

[20] Souza GS, de Carvalho LP, de Melo EJT, da Silva FCV, Machado OLT, Gomes VM (2019). A synthetic peptide derived of the β2–β3 loop of the plant defensin from Vigna unguiculata seeds induces Leishmania amazonensis apoptosis-like cell death. Amino Acids.

[21] Shelenkov A, Slavokhotova A, Odintsova T (2020). Predicting antimicrobial and other cysteine-rich peptides in 1267 plant Transcriptomes. Antibiotics..

[22] The United States Centers for Disease Control and Prevention (2019). Antibiotic Resistance Threats in the United States.

[23] Chou S, Wang J, Shang L, Akhtar MU, Wang Z, Shi B (2019). Short, symmetric-helical peptides have narrow-spectrum activity with low resistance potential and high selectivity. Biomater Sci.

[24] Helander IM, Mattila-Sandholm T (2000). Fluorometric assessment of gram-negative bacterial permeabilization. J Appl Microbiol.

[25] Huang D, Boxin OU, Prior RL (2005). The chemistry behind antioxidant capacity assays. J Agric Food Chem.

[26] Girardello R, Bispo PJM, Yamanaka TM, Gales AC (2012). Cation concentration variability of four distinct Mueller-Hinton agar brands influences polymyxin B susceptibility results. J Clin Microbiol.

[27] Santos JS, Alvarenga Brizola VR, Granato D (2017). High-throughput assay comparison and standardization for metal chelating capacity screening: a proposal and application. Food Chem.

[28] Pechous RD, Broberg CA, Stasulli NM, Miller VL, Goldman WE (2015). In vivo transcriptional profiling of yersinia pestis reveals a novel bacterial mediator of pulmonary inflammation. MBio..

[29] Fleeman RM, Macias LA, Brodbelt JS, Davies BW (2020). Defining principles that influence antimicrobial peptide activity against capsulated Klebsiella pneumoniae. Proc Natl Acad Sci U S A.

[30] Joo HS, Fu CI, Otto M (2016). Bacterial strategies of resistance to antimicrobial peptides. Philos Trans R Soc B Biol Sci.

[31] Chen HD, Groisman EA (2013). The biology of the PmrA/PmrB two-component system: the major regulator of lipopolysaccharide modifications. Annu Rev Microbiol.

[32] Simpson BW, Trent MS (2019). Pushing the envelope: LPS modifications and their consequences. Nat Rev Microbiol.

[33] Rubin EJ, Herrera CM, Crofts AA, Trent MS (2015). PmrD is required for modifications to escherichia coli endotoxin that promote antimicrobial resistance. Antimicrob Agents Chemother.

[34] Yan A, Guan Z, Raetz CRH (2007). An undecaprenyl phosphate-aminoarabinose flippase required for polymyxin resistance in Escherichia coli. J Biol Chem.

[35] Hagiwara D, Yamashino T, Mizuno T (2004). A genome-wide view of the Escherichia coli BasS-BasR two-component system implicated in iron-responses. Biosci Biotechnol Biochem.

[36] Wösten MMSM, Kox LFF, Chamnongpol S, Soncini FC, Groisman EA (2000). A signal transduction system that responds to extracellular iron. Cell..

[37] Alexander JL, Thompson Z, Cowan JA (2018). Antimicrobial Metallopeptides. ACS Chem Biol.

[38] Bleackley MR, Vasa S, Harvey PJ, Shafee TMA, Kerenga BK, Soares da Costa TP (2020). Histidine-rich defensins from the solanaceae and brasicaceae are antifungal and metal binding proteins. J Fungi.

[39] Mirakhorli N, Norolah Z, Foruzandeh S, Shafizade F, Nikookhah F, Saffar B (2019). Multi-function plant defensin, antimicrobial and heavy metal adsorbent peptide. Iran J Biotechnol.

[40] Meindre F, Lelie D, Loth K, Mith O, Aucagne V, Berthomieu P (2014). The nuclear magnetic resonance solution structure of the synthetic AhPDF1.1b plant defensin evidences the structural feature within the γ-motif. Biochemistry..

[41] Blahut M, Sanchez E, Fisher CE, Outten FW (1867). Fe-S cluster biogenesis by the bacterial Suf pathway. Biochim Biophys Acta, Mol Cell Res.

[42] Iverson TM, Luna-Chavez C, Cecchini G, Rees DC (1999). Structure of the *Escherichia coli* fumarate reductase respiratory complex. Science..

[43] Ollagnier-de Choudens S, Loiseau L, Sanakis Y, Barras F, Fontecave M (2005). Quinolinate synthetase, an iron-sulfur enzyme in NAD biosynthesis. FEBS Lett.

[44] McHugh JP, Rodríguez-Quiñones F, Abdul-Tehrani H, Svistunenko DA, Poole RK, Cooper CE (2003). Global iron-dependent gene regulation in Escherichia coli: a new mechanism for iron homeostasis. J Biol Chem.

[45] Folsom JP, Parker AE, Carlson RP (2014). Physiological and proteomic analysis of Escherichia coli iron-limited chemostat growth. J Bacteriol.

[46] Andrews SC, Robinson AK, Rodríguez-Quiñones F (2003). Bacterial iron homeostasis. FEMS Microbiol Rev.

[47] Ling J, Pan H, Gao Q, Xiong L, Zhou Y, Zhang D (2013). Aerobactin synthesis genes iucA and iucC contribute to the pathogenicity of avian pathogenic Escherichia coli O2 strain E058. PLoS One.

[48] Noinaj N, Guillier M, Barnard TJ, Buchanan SK (2010). TonB-dependent transporters: regulation, structure, and function. Annu Rev Microbiol.

[49] Leebens-Mack JH, Barker MS, Carpenter EJ, Deyholos MK, Gitzendanner MA, Graham SW (2019). One thousand plant transcriptomes and the phylogenomics of green plants. Nature..

[50] Matasci N, Hung LH, Yan Z, Carpenter EJ, Wickett NJ, Mirarab S (2014). Data access for the 1,000 plants (1KP) project. Gigascience..

[51] Shelenkov AA, Slavokhotova AA, Odintsova TI (2018). Cysmotif searcher pipeline for antimicrobial peptide identification in plant Transcriptomes. Biochem Mosc.

[52] Madeira F, Park YM, Lee J, Buso N, Gur T, Madhusoodanan N (2019). The EMBL-EBI search and sequence analysis tools APIs in 2019. Nucleic Acids Res.

[53] Simon MD, Heider PL, Adamo A, Vinogradov AA, Mong SK, Li X (2014). Rapid flow-based peptide synthesis. ChemBioChem..

[54] Moyer TB, Heil LR, Kirkpatrick CL, Goldfarb D, Lefever WA, Parsley NC (2019). PepSAVI-MS reveals a Proline-rich antimicrobial peptide in *Amaranthus tricolor*. J Nat Prod.

[55] Kirkpatrick CL, Broberg CA, McCool EN, Lee WJ, Chao A, McConnell EW (2017). The “PepSAVI-MS” pipeline for natural product bioactive peptide discovery. Anal Chem.

[56] Smythers AL, McConnell EW, Lewis HC, Mubarek SN, Hicks LM (2020). Photosynthetic metabolism and nitrogen reshuffling are regulated by reversible cysteine thiol oxidation following nitrogen deprivation in chlamydomonas. Plants..

[57] Käll L, Canterbury JD, Weston J, Noble WS, MacCoss MJ (2007). Semi-supervised learning for peptide identification from shotgun proteomics datasets. Nat Methods.

[58] Gianetto QG (2018). imp4p: Imputation for Proteomics.

[59] Benjamini Y, Hochberg Y (1995). Controlling the false discovery rate: a practical and powerful approach to multiple testing. J R Stat Soc B.

[60] Mi H, Ebert D, Muruganujan A, Mills C, Albou LP, Mushayamaha T (2021). PANTHER version 16: a revised family classification, tree-based classification tool, enhancer regions and extensive API. Nucleic Acids Res.

[61] Vizcaíno JA, Csordas A, del Toro N, Dianes JA, Griss J, Lavidas I (2016). 2016 update of the PRIDE database and its related tools. Nucleic Acids Res.

