# Supplementary material for: Proteomic response of Escherichia coli to a membrane lytic and iron chelating truncated Amaranthus tricolor defensin
Source: BMC Microbiol. 2021 Apr 12;21:110. doi: 10.1186/s12866-021-02176-4 (PMC8042948; doi:10.1186/s12866-021-02176-4)
Supplement: Supplementary file 2 — Additional file 2: Figures S1 and S2. Figures illustrating alignment of defensins predicted from the transcriptome of A. tricolor (Fig. S1) and the IC50 of Atr-DEF2(G39-C54) against E. coli 25922 and K. pneumoniae VK148 (Fig. S2). [file 12866_2021_2176_MOESM2_ESM.docx]

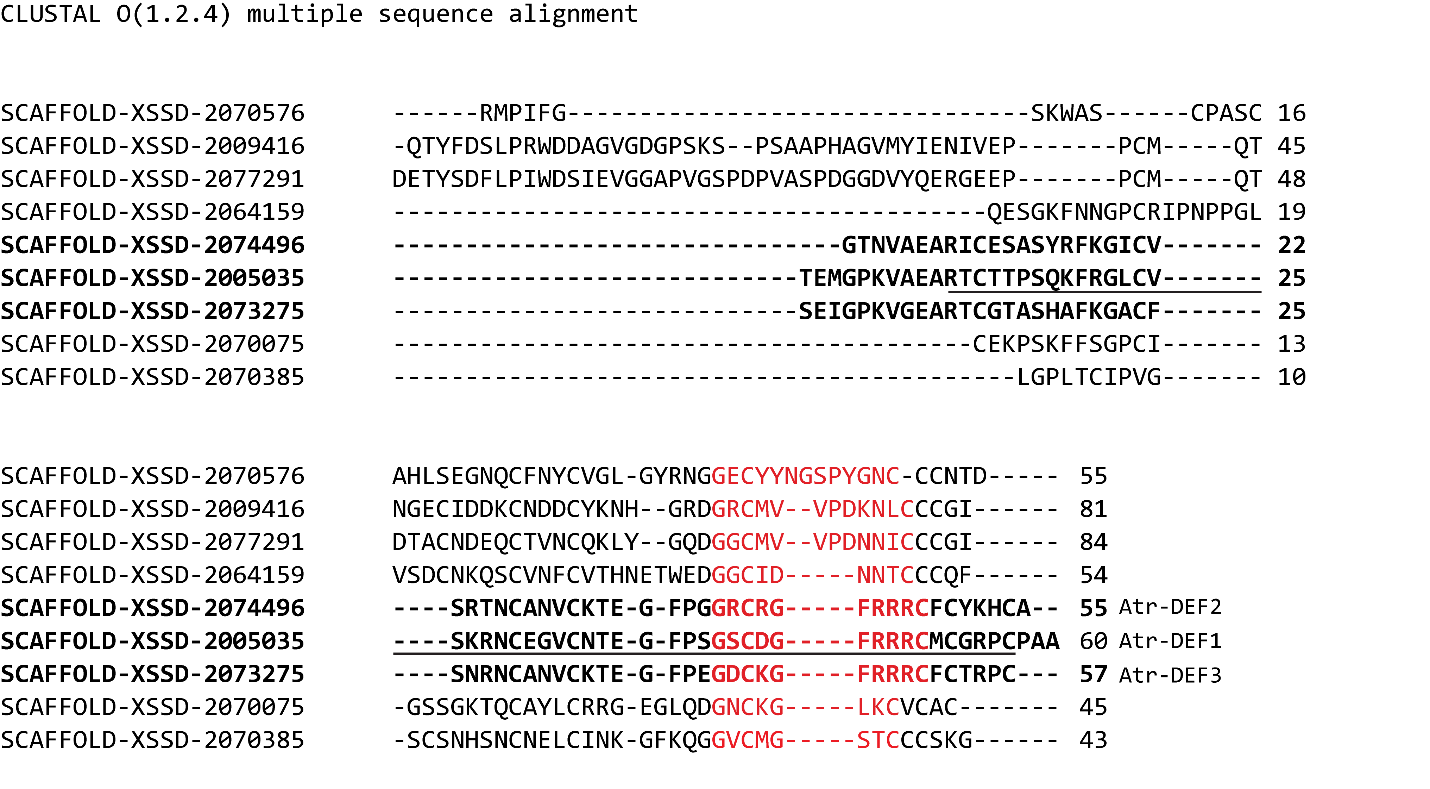


Figure S1. Predicted mature sequences of defensins identified within the transcriptome of *A. tricolor* which contain a canonical γ-core motif (GXCX_3‐9_C, where X_n_ is the number of residues between cysteines) (red). The experimentally determined mature sequence of Atr-DEF1 is underlined.


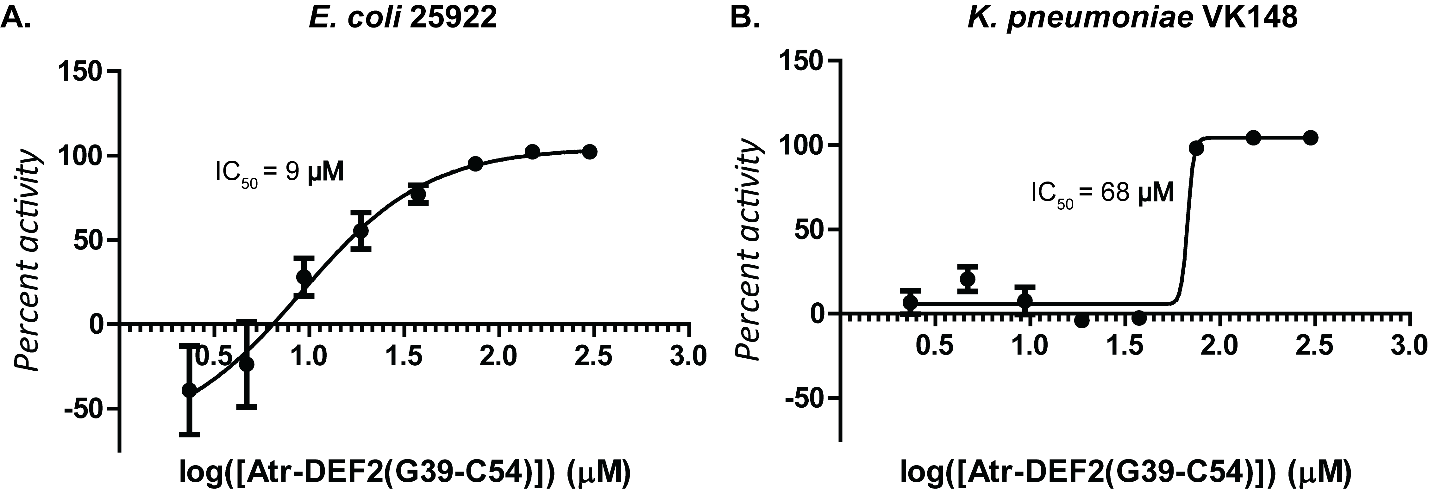


Figure S2. IC_50_ of Atr-DEF2(G39-C54) against (A) *E. coli* 25922 and (B) *K. pneumoniae* VK148.
